# Supplementary material for: Trends in Norovirus Genotypes in South Korea, 2019–2024: Insights from Nationwide Dual Typing Surveillance
Source: Viruses. 2025 Nov 30;17(12):1572. doi: 10.3390/v17121572 (PMC12737402; doi:10.3390/v17121572)
Supplement: Supplementary file 1 [file viruses-17-01572-s001.zip › viruses-3939763-supplementary.pdf]

viruses

MDPI

**Supplementary Table S1.** Regional distribution of norovirus-positive samples and detection rates among acute gastroenteritis cases, South Korea during 2019–2024. Data are presented as number positive/total tested (%). Regional variations highlight geographic heterogeneity in detection rates across provinces.

| Region | 2019 | 2020 | 2021 | 2022 | 2023 | 2024 | Total |
|---|---|---|---|---|---|---|---|
| Seoul | 29/238 (12.2) | 16/136 (11.8) | 13/110 (11.8) | 23/99 (23.2) | 16/67 (23.9) | 22/109 (20.2) | 119/759 (15.7) |
| Busan | 22/614 (3.6) | 13/422 (3.1) | 6/356 (1.7) | 29/541 (5.4) | 13/566 (2.3) | 11/541 (2.0) | 94/3040 (3.1) |
| Daegu | 34/417 (8.6) | 10/329 (3.0) | 71/519 (13.7) | 29/446 (6.5) | 1/472 (0.2) | 10/403 (2.5) | 155/2586 (6.1) |
| Incheon | 96/821 (11.7) | 43/753 (5.7) | 63/843 (7.5) | 49/805 (6.1) | 55/819 (6.7) | 38/777 (4.9) | 344/4818 (7.1) |
| Gwangju | 220/873 (25.2) | 233/1123 (20.7) | 365/1408 (25.9) | 506/1793 (28.2) | 424/1850 (22.9) | 345/1515 (22.8) | 2093/8562 (24.4) |
| Daejeon | 20/940 (2.1) | 14/925 (1.5) | 130/572 (22.7) | 39/510 (7.6) | 87/574 (15.2) | 152/635 (23.9) | 442/4156 (10.6) |
| Ulsan | 17/445 (3.8) | 7/285 (2.5) | 8/250 (3.2) | 8/390 (2.1) | 27/347 (7.8) | 64/395 (16.2) | 131/2112 (6.2) |
| Sejong* | - | - | - | 10/291 (3.4) | 25/618 (4.0) | 22/299 (7.4) | 57/1208 (4.7) |
| Gyeonggi-do | 89/863 (10.3) | 27/415 (6.5) | 26/240 (10.8) | 23/247 (9.3) | 33/283 (11.7) | 34/230 (14.8) | 232/2278 (10.2) |
| Gangwon-do | 63/611 (10.3) | 21/1350 (1.6) | 80/1533 (5.2) | 114/1638 (7.0) | 74/1789 (4.1) | 90/1180 (7.6) | 442/8101 (5.5) |
| Chungcheong-buk-do | 7/734 (1.0) | 14/554 (2.5) | 31/655 (4.7) | 10/579 (1.7) | 8/647 (1.2) | 9/598 (1.5) | 79/3767 (2.1) |
| Chungcheong-nam-do | 39/507 (7.7) | 29/134 (21.6) | 3/251 (1.2) | 9/391 (2.3) | 8/617 (1.3) | 5/522 (1.0) | 93/2422 (3.8) |
| Jeollabuk-do | 78/801 (9.7) | 47/752 (6.3) | 48/670 (7.2) | 17/645 (2.6) | 57/690 (8.3) | 45/723 (6.2) | 292/4281 (6.8) |
| Jeollanam-do | 30/536 (5.6) | 39/556 (7.0) | 50/410 (12.2) | 51/875 (5.8) | 198/2002 (9.9) | 217/2058 (10.5) | 585/6437 (9.1) |
| Gyeongsang-buk-do | 29/631 (4.6) | 15/499 (3.0) | 36/877 (4.1) | 86/1011 (8.5) | 59/925 (6.4) | 41/663 (6.2) | 266/4606 (5.8) |
| Gyeongsang-nam-do | 23/508 (4.5) | 4/543 (0.7) | 17/633 (2.7) | 17/541 (3.1) | 20/888 (2.3) | 29/822 (3.5) | 110/3935 (2.8) |
| Jeju-do | 21/674 (3.1) | 24/604 (4.0) | 19/779 (2.4) | 18/753 (2.4) | 25/732 (3.4) | 9/547 (1.6) | 116/4089 (2.8) |
| Total | 817/10213 (8.0) | 556/9380 (5.9) | 966/10106 (9.6) | 1038/11555 (9.0) | 1130/13886 (8.1) | 1143/12017 (9.5) | 5650/67157 (8.4) |

* Data collection in Sejong was initiated in August 2022 as part of the EnterNet-Korea surveillance program.
